# Failure to Demonstrate That Playing Violent Video Games Diminishes Prosocial Behavior

**DOI:** 10.1371/journal.pone.0068382

**Published:** 2013-07-03

**Authors:** Morgan J. Tear, Mark Nielsen

**Affiliations:** School of Psychology, The University of Queensland, St. Lucia, Queensland, Australia; George Mason University/Krasnow Institute for Advanced Study, United States of America

## Abstract

**Background:**

Past research has found that playing a classic prosocial video game resulted in heightened prosocial behavior when compared to a control group, whereas playing a classic violent video game had no effect. Given purported links between violent video games and poor social behavior, this result is surprising. Here our aim was to assess whether this finding may be due to the specific games used. That is, modern games are experienced differently from classic games (more immersion in virtual environments, more connection with characters, etc.) and it may be that playing violent video games impacts prosocial behavior only when contemporary versions are used.

**Methods and Findings:**

Experiments 1 and 2 explored the effects of playing contemporary violent, non-violent, and prosocial video games on prosocial behavior, as measured by the pen-drop task. We found that slight contextual changes in the delivery of the pen-drop task led to different rates of helping but that the type of game played had little effect. Experiment 3 explored this further by using classic games. Again, we found no effect.

**Conclusions:**

We failed to find evidence that playing video games affects prosocial behavior. Research on the effects of video game play is of significant public interest. It is therefore important that speculation be rigorously tested and findings replicated. Here we fail to substantiate conjecture that playing contemporary violent video games will lead to diminished prosocial behavior.

## Introduction

Video games proliferate most contemporary Western cultures and are one of the most commercially consumed forms of media. Indeed, major titles in the video game category regularly outsell the most successful titles in other media formats. For example, according to US and UK sales, the highest grossing video game over five days through to the end of 2011 was Call of Duty: Modern Warfare 3 ($775 m). This eclipses the highest grossing movie over a similar period in 2010, The Dark Knight ($20 c). However, not only are video games pervasive throughout contemporary culture, they are typically violent in nature and have thus become a target of public concern. Indeed, it is common for the behavior of those perpetuating extreme acts of violence, such as that by Anders Breivik, James Holmes, and Adam Lanza, to be linked to video game play (though this link is often one of public perception: video games have been falsely attributed in some similar cases [1]). Spurred by this public concern, the past two decades have seen a concerted effort devoted to understanding whether a link between violent video games and real-world behavior exists.

Because of their violent nature, the vast majority of research into video games has focused on the way game play impacts anti-social behavior. A recent meta-analysis conducted by Anderson and his colleagues suggested that violent video games increase anti-social behavior [2]. However, the value of that meta-analysis is debated [3], [4], reflecting a wider debate in the literature (see [5] for a summary). Regardless of which theoretical camp is right, comparatively little research has explored the effects of video games on other outcomes. Prosocial behavior is one such example. If playing violent video games increases anti-social behavior it seems reasonable to expect playing will also diminish prosocial behavior. There is some evidence to support this. Participants who played a violent game, compared to a non-violent game, have been reported to be less likely to cooperate [6], and less likely to reward a confederate [7]. Conversely, studies from two camps of researchers demonstrated that violent video games can even increase prosocial behavior [8], [9]. Moreover, the impact of playing violent video games is highlighted by findings that playing prosocial games can increase helping behavior and decrease aggressive outcomes [10]. Few studies, however, have directly contrasted the effects of violent and prosocial video games on prosocial behavior.

In a recent noteworthy article, Greitemeyer and Osswald [11] demonstrated that video games can have beneficial effects on behavior, provided the games have prosocial content. Participants played a classic prosocial game (Lemmings, where players must save as many game characters as possible), a classic violent game (Lamers, where players must kill all the characters as quickly as possible), or a classic neutral game (Tetris, where players must arrange shapes to fit together) for 8 minutes and then rated the game on measures of enjoyment. Following gameplay, participants were presented with the pen-drop test [12], [13], where the experimenter accidentally spills some pens onto the floor. Whether the participant helps gather the pens or not is taken as a measure of spontaneous, unrequested assistance. Significantly more participants who played the prosocial game helped gather the pens (67%) than participants who played the violent game (28%) or the neutral game (33%). That is, those who played the prosocial game were more inclined to help pick up the pens. Notably, there was no significant difference between participants who played the violent and neutral games.

There are several explanations for Greitemeyer and Osswald’s [11] failure to find an effect of playing violent video games on prosocial behavior. One is that participants played games for 8 minutes. This interval may not be long enough to elicit an effect. Indeed, most research with violent video games uses a playing time of 15 minutes or more. Greitemeyer and Osswald themselves comment that longer exposure times should reveal significant differences (p.215). Furthermore, the ‘classic’ video games they used may not have been strong enough. Contemporary games are demonstrably more immersive [14], realistic [15], and violent [16], and subsequently require more emotional investment. Modern video game stimuli also vary in terms of competitiveness and difficulty [17], and the underlying intentions motivating game play [18]. Indeed, Greitemeyer [19] speculated that “Modern, graphically sophisticated games may be more involving and thus should affect helping behavior to a greater extent” (p.252). Moreover, given public concern, the applied value of using contemporary and, importantly, commercially available video games is potentially more informative and valuable. If violent video games impact on prosocial tendencies we need to know if the games people currently play have this effect.

The current set of experiments was designed to explore whether contemporary violent video games lead to decreases in prosocial behavior. Thus, the aim in Experiment 1 was to extend Greitemeyer and Osswald [11] using longer exposure times and contemporary video games as stimuli. We included the anti-social video game Grand Theft Auto as our main game of interest, but to assess whether the anti-social nature of the game or the portrayed violence is more important for reducing prosocial behavior, we included Call of Duty as a violent control. We compared these two violent games to a non-violent and a prosocial video game.

## Experiment 1

Participants were exposed to one of four different types of video games: anti-social, violent, non-violent, or prosocial. It was hypothesized that, using contemporary exemplars of video games, prosocial behavior would be higher in participants who played a prosocial video game and lower in participants who played the anti-social or violent video game.

### Method

#### Participants

Sixty-four undergraduate students (56% male) at a large metropolitan university (age range 17–33, *M* = 20.30, *SD* = 3.61) took part in Experiment 1for course credit. Participants were mostly Caucasian (88%) with a minority reporting Asian ethnicity (12%). Participants gave written informed consent to participate in the experiment. Ethical clearance was granted by the Behavioural & Social Sciences Ethical Review Committee at the University of Queensland.

#### Video games

We note here that it is difficult to dichotomize games as either solely violent or prosocial since many violent games include prosocial themes (e.g. killing villains to save the world, see [9]). We tried to circumvent this by including games where players could engage in only violent or prosocial actions (e.g. killing zombies/attacking police vs. taking care of an animal). Participants played one of the following four video games:

#### 1) Anti-social (grand theft auto IV)

Grand Theft Auto is an open-world sandbox game, meaning participants can adopt a non-linear style of playing and explore their environment. To ensure participants engaged in aggressive behaviors in the game we made all the in-game weapons (e.g. handguns, rifles, rocket launchers, etc.) available at the start of the session. Grand Theft Auto was included as an exemplar of an anti-social game. Since, intent is an important component of the standard definition of aggression [20], [21], we distinguish between styles of violence; morally defensible and indefensible violence. When playing the violent game exemplar described below (Call of Duty), players engaged in violent acts to preserve the lives of themselves and others (morally defensible). In Grand Theft Auto, however, players engaged in violence towards other members of a society often for no defensible reason, for example, stealing cars, damaging property, running over innocent civilians, running away from and killing police. The intent of this violence is not related to self-preservation or any other in-game objectives, it is entirely for the sake of being violent (morally-indefensible).

#### 2) Violent (call of duty: black ops)

Call of Duty was selected as a violent control game. In Call of Duty, which is a first-person shooter game, players assumed the role of various soldiers who wield firearms and explosives, and can engage in close quarters combat. Participants played the ‘zombie’ mode, where they needed to simultaneously solve puzzles to progress through a series of rooms, while also killing zombies with a variety of guns and weapons. As previously mentioned, the violence in Call of Duty reflects a morally defensible intent to survive, or avoid death. Many games that could be considered violent employ a similar style of ‘self-defense violence’ (killing others to avoid being killed). To this end, Call of Duty served as a violent control to the deliberately anti-social content of Grand Theft Auto. Call of Duty qualified as a violent game because the zombie deaths were often quite extreme and grotesque (e.g. zombie corpses could be blown apart). We also selected the zombie mode because the gameplay was reasonably linear, meaning that each participant had a similar experience while playing the game.

#### 3) Prosocial (world of zoo)

In World of Zoo players needed to create a successful zoo exhibit, which was achieved by taking care of animals by feeding, cleaning, and playing with them. Unlike the other games described here, World of Zoo is not explicitly marketed towards adults. It is, however, one of the few commercially available games that requires prosocial behavior and does not contain violent or adult themes.

#### 4) Non-violent (portal 2)

This is a non-violent puzzle game where the player used a gun that shoots entry and exit points of a portal, allowing them to access areas they would not normally be able to. This game acted as a non-violent control condition because, like Call of Duty, the player uses a gun-shaped tool to interact with the virtual world. The gun in Portal 2, however, shoots portals instead of bullets.

#### Procedure and design

Participants were informed that the session would comprise two unrelated studies that were bundled together in the interest of time. The first study was described as a pilot study that asked for participants’ reactions to pre-selected games so we could assess their suitability as stimuli for future experiments. Participants were randomly assigned to play one of the four previously outlined games for 20 minutes and then asked to fill out a questionnaire ostensibly aimed at assessing their experience of playing the game. This questionnaire contained questions regarding the participant’s levels of interest, frustration, and arousal experienced, and was used to test whether the games differed on factors other than the presence or absence of violent content. The questionnaire contained 12 Likert items (1: strongly disagree, to 9: strongly agree, example items: the game was too hard, the game got my heart racing, the game kept my attention). The second study was described as a distinct investigation of social attitudes. To this end, participants were asked to complete a series of questionnaires unrelated to the current study to reinforce the ostensible reason for the second study and to minimize suspicion about the true hypothesis. Once participants finished filling out these questionnaires the experimenter said he had to rush to the other side of campus for an ostensible appointment he had forgotten, and that he would debrief the participant via email. The experimenter gathered his belongings, namely some folders, some pens balanced on the folders, and a cup of coffee, all to emphasize that he was fully laden. He then opened the door and, as the participant moved past the experimenter, ‘accidentally’ tipped the folders such that the pens fell to the floor, muttering under his breath as he did so. As per Greitemeyer and Osswald [11], the experimenter waited five seconds for the participants to help pick up the pens. The participant was considered to have acted prosocially if he/she helped pick up at least one pen (some pens would land in a way that would not make sense for the participant to pick up). Once the pens had been gathered and the participant was on his/her way, the experimenter called the participant back into the room where he/she was probed for suspicion and debriefed. Neither in this experiment nor any of the subsequent experiments did any participant report suspecting the true aim of the experiment. Similarly, here, and in all subsequent experiments, the experimenter was not blind to experimental conditions. For the sake of brevity we do not mention this again.

### Results and Discussion

Table 1 shows the number of participants who helped (and did not help) by condition. Here it can be seen that there are small differences in prosocial behavior between conditions. A Chi-Square analysis was unfeasible given that some cells contained less than 5 cases. We opted to use Fisher’s Exact Tests to determine the feasibility of collapsing across similar conditions. First, we compared the games with violence (anti-social vs. violent, *p* = .220, two-tailed) and the games without violence (prosocial vs. non-violent, *p* = .685, two-tailed). Since there was no difference between similar games, and in order to increase power, we then collapsed the conditions into two broad categories (Violence-Present vs. Violence-Absent) and calculated a Chi-Square. The test on the collapsed data found no difference in prosocial behavior between Violence-Present games and the Violence-Absent games, *χ* ^2^ (1, *N* = 64) = 0.00, *p* = 1.000, V = 0.00.

**Table 1:** Frequency of prosocial behavior across video game conditions in Experiment 1. Percentages in parentheses.

	Behavioral Outcome	
Video Game Condition	Help	No Help
Anti-social	2 (12.5)	14 (87.5)
Violent	6 (37.5)	10 (62.5)
Prosocial	3 (18.8)	13 (81.3)
Non-violent	5 (31.3)	11 (68.8)

For all experiments we report whether the self-report measures differed according to levels of the video game type variable, and follow up significant differences with hierarchical logistic regressions to determine if the self-report measures predict variance above and beyond video game type.

In experiment 1, a series of one-way ANOVAs established that our video game stimuli differed beyond presence or absence of violence. Analyses revealed a main effect of self-reported frustration, *F*(1, 3) = 3.72, *p* = .016. Follow-up analyses showed that the violent game was significantly more frustrating (*M* = 5.13, *SD* = 1.60) than the non-violent game (*M* = 3.52, *SD* = 1.47) and the prosocial game (*M* = 3.69, *SD* = 1.05), ps<.010,but not the anti-social game (*M* = 4.30, *SD* = 1.81), *p* = .126.

There was also a main effect of self-reported arousal, *F*(1, 3) = 13.23, *p*<.001. The prosocial game was significantly less arousing (*M* = 3.30, *SD* = 1.40) than all of the other games; violent (*M* = 6.03, *SD* = 1.17), anti-social (*M* = 5.36, *SD* = 1.64), and non-violent (*M* = 5.63, *SD* = 1.09), *p*s<.001.

Finally, the games differed on self-reported interest, *F*(1, 3) = 14.96, *p*<.001. Participants found the non-violent game significantly more interesting (*M* = 7.59, *SD* = 0.92) than all of the other games; violent (*M* = 6.09, *SD* = 1.66), anti-social (*M* = 6.15, *SD* = 1.02), and pro-social (*M* = 5.26, *SD* = 1.53), *p*s<.003.

We conducted a hierarchical logistic regression to determine if the self-report variables could account for additional variance in prosocial behavior above and beyond the video game manipulation. We entered the video game variable at Step 1 and the three self-report measures at Step 2. As a set of predictors, the self-report measures do not account for additional variance above and beyond the video game manipulation, Nagelkerke *R* ^2^ = .13, *χ* ^2^(3, *N* = 64) = 2.42, *p* = .491. None of the three self report measures were significantly linked to prosocial behavior, Wald tests<.88, *p*s>.348.

Greitemeyer and Osswald [11] previously demonstrated that playing a prosocial video game led participants to be more likely to engage in spontaneous, unrequested helping behavior whereas playing a violent game showed no impact. Here we were unable to replicate this finding of improved performance for participants in the prosocial game condition. Moreover, despite extending the playing time to 2 ins and using commercially available, contemporary games, we also failed to show a reduction in prosocial behavior from playing violent games. An initial interpretation of our results might suggest we have simply found a baseline rate of helping in our population. Studies using the pen-drop task report baseline rates of around 30% of participants helping to pick up the pens [11], [13]. It is thus possible that our stimuli were not potent enough to elicit a primed response. We find this unlikely given that effects of violent games have been shown with much simpler games [11], [22]–[24]. Furthermore, the entire basis for using contemporary games over classic games is that they typically offer a much more enriched experience so intuition would posit the effect should be stronger for contemporary games. An alternative explanation is the timing of the pen-drop task. In order to avoid arousing suspicion from participants we inserted other tasks between game play and the test for them to complete. This may have inadvertently biased our protocol against revealing an effect by diluting the impact of the games (or by removing blatant demand characteristics). We attempted to remedy this in Experiment 2.

The prosocial game World of Zoo was marketed primarily as a children’s game and, thus, inherently differed from the other games in more ways than just the presence of violent/prosocial content (e.g. significantly less arousing), a problem acknowledged in other research [17], [18]. Since we are interested in why Greitemeyer and Osswald [11] could not show a detrimental effect of violent games beyond non-violent controls, we decided to omit World of Zoo (the prosocial game) from subsequent testing. Further, in Experiment 1we included Call of Duty to control for the type of violent content (morally defensible vs. morally indefensible). As there was no statistical difference in performance between Call of Duty and Grand Theft Auto we omitted the former in subsequent testing.

## Experiment 2

In Experiment 1, participants filled out questionnaires directly after playing the game, whereas in Greitemeyer and Osswald [11] the pen-drop task happened immediately following play. The filler questionnaires in Experiment 1 took anywhere between five and ten minutes, and past literature has shown that filler tasks can nullify the violent video game effect [25], though it could also be argued that filler tasks remove blatant demand characteristics in violent video game studies. Further, the pens were dropped as participants left the room, whereas Greitemeyer and Osswald did so half-way through the experimental session, necessitating further interaction between the participant and experimenter. In Experiment 2 we, therefore, manipulated the administration of the pen-drop task to bring the test phase closer to the video game prime by either feigning the end of the session or by administering the pen-drop during the middle of the session.

### Method

#### Participants

We recruited 64 undergraduate participants (55% male) from the first-year participant pool at a large metropolitan university. Participant ages ranged from 17–43 (*M* = 21.63, *SD* = 5.50). Most were Caucasian (77%), though some reported Asian ethnicity (14%), or other (9%). Participants provided written informed consent and either received course credit for participating in the experiment or a small monetary reimbursement. Ethical clearance was granted by the Behavioural & Social Sciences Ethical Review Committee at the University of Queensland.

#### Video games

Given our continued focus on using contemporary games to demonstrate the violent video game effect and the difficulty we had in procuring an adult-oriented prosocial video game, we only used two games in Experiment 2; Grand Theft Auto (anti-social) and Portal 2 (non-violent).

#### Procedure and design

As per Experiment 1, participants were instructed that the experimental session comprised two ostensibly unrelated studies; the first to gather participants’ opinions of pre-selected games to determine whether they would be suitable stimuli for a future experiment, and the second to pilot test various measures of social attitude. Participants were randomly allocated to play either Grand Theft Auto or Portal 2for 20 minutes. Following the game, and to keep the story of the first ostensible study believable, the participant rated the games on the same measures as in Experiment 1. This was the only task the participant completed between the video game and pen-drop task, taking no more than 60 seconds (12 items). We deemed this short questionnaire necessary for the cover-story of the first study.

We then manipulated the context in which the pen-drop task was administered; having it follow the video game prime and ending the session there (Session-Ends), or following the video game prime but with the session continuing into the second ostensible experiment (Session-Continues).

In the Session-Ends condition, following the participant completing the ostensible first study, the experimenter ‘realized’ that he forgot to bring the materials for the second study and would have to end the experimental session early. As in Experiment 1, the experimenter gathered his belongings, making him appear sufficiently laden, and opened the door for the participant. The experimenter dropped the pens as the participant moved past him, waiting for the participant to help gather the pens. Participants were then probed for suspicion and debriefed.

In the Session-Continues condition, participants finished the questionnaire for the ostensible first study and handed it to the experimenter. The experimenter then reached for the materials for the ostensible second study, knocking over a tin of pens placed at the end of a table, equidistant from both the experimenter and participant. The experimenter waited for the participant’s reaction; did they pick up the pens or not? Once the pens had been gathered the experimenter began the second study. Participants completed the second study, were probed for suspicion, and then debriefed.

### Results and Discussion

Table 2 shows the rates of helping (or not) for both games across both timing conditions. To investigate the effect of the relative timing of the pen-drop we conducted a Chi-Square analysis to assess whether there was a main effect of timing (Session-Ends vs. Session-Continues). The test showed that a greater proportion of participants helped when the pens spilled in the middle of the experimental session compared to the proportion who helped when the experimental session was thought to end, *χ* ^2^ (1, *N* = 64) = 12.30, *p*<.001, *V* = .438.

**Table 2:** Frequency of prosocial behavior across video game and timing conditions in Experiment 2.

		Behavioral Outcome	
Timing Condition	Video Game Condition	Help	No Help
Session-Ends	Anti-social	6 (37.5)	10 (62.5)
	Non-violent	4 (25.0)	12 (75.0)
Session-Continues	Anti-social	14 (87.5)	2 (12.5)
	Non-violent	10 (62.5)	6 (37.5)

Percentages in parentheses.

Within each level of the timing variable we conducted logistic regressions to see whether the rate of helping differed according to the game played. The type of video game played did not have an effect in either the Session-Ends condition (anti-social: 38%, non-violent: 25%), Nagelkerke *R* ^2^ = .03, *χ* ^2^(1, *N* = 32) = 0.59, *p* = .444 or the Session-Continues condition (anti-social: 87%, non-violent: 63%), Nagelkerke *R* ^2^ = .12, *χ* ^2^ (1, *N* = 32) = 2.76, *p* = .096.

We ran t-tests to determine if the video games were experienced differently. Between the two games, we found no differences in terms of frustration (*t*(62) = 0.13, p = .895), arousal (*t*(62) = 1.04, p = .300), or interest (*t*(62) = 0.50, *p* = .617).

It appears that neither arrangement of the pen-drop task in Experiment 2 was sufficient to elicit the violent video game effect. There was no main effect of game type: the number of participants helping was statistically equal across the two games (anti-social: 63%; non-violent: 44%). There was, however, a main effect of the context in which the pen-drop was administered: rate of helping was significantly higher in the Session-Continues condition (75%) than in the Session-Ends condition (31%).

In order to account for possible lack of power given the relatively small cell sizes used here and in Experiment 1, we collapsed the Session-Ends data with the anti-social and non-violent data from Experiment 1. Despite doubling participant numbers, differences in rates of helping between anti-social and non-violent conditions remained non-significant, *χ* ^2^ (1, *N* = 64) = 0.80, *p* = .777, *V* = .035.

Experiment 2 was designed to evaluate whether manipulating the contextual administration of the pen-drop task would help reveal an effect of playing an anti-social game. While subtle contextual differences in the administration of the pen-drop task are able to move base-rates of helping behavior, they were not sufficient for revealing the anticipated violent video game effect. Given the failure to show an effect of violent video games, it was necessary to attempt a procedural replication of Greitemeyer and Osswald [11].

## Experiment 3

In Experiments 1 and 2 we failed to find any effect of playing a violent video game on prosocial behavior. It is conceivable that the motive for violence in classic games is much less ambiguous than contemporary games (e.g. killing creatures to prevent them for winning is more obviously violent than killing enemies to win a politicized and emotional war). Thus, we decided to replicate Greitemeyer and Osswald [11], using classic games (Lamers and Lemmings), decreasing our exposure time to 8 minutes, and administering the pen-drop in the Session-Continues style of Experiment 2.

### Method

#### Participants

We recruited 32 undergraduate participants (66% male) from the first-year participant pool at a large metropolitan university. Participant ages ranged from 17–26 (M = 19.5, SD = 2.29). Again, most were Caucasian (81%), with small minorities reporting Asian ethnicity (16%), or other (3%). Participants provided written informed consent and received course credit for participating in the experiment. Ethical clearance was granted by the Behavioural & Social Sciences Ethical Review Committee at the University of Queensland.

#### Video games

Following Greitemeyer and Osswald [11], participants in Experiment 3were randomly assigned to play one of the following two games:

#### 1) Prosocial (lemmings)

The general prosocial aim of this game is to prevent a colony of lemmings from mindlessly marching over cliff edges or into hazards by assigning them useful roles (e.g. assigning a parachute role so they do not fall to their death). This gets progressively more taxing as players proceed through the increasingly difficult levels. A player’s score is determined by how many lemmings they save from mindless self-death.

#### 2) Violent (lamers)

Lamers is a violent parody of Lemmings, with the goal being to kill as many of the characters as possible before they reach their goal. Players have various weapons at their disposal, including guns and explosives.

#### Procedure and design

Following Greitemeyer and Osswald [11], and as per Experiment 2, we administered the pen-drop in the Session-Continues form. Again, this experiment was run as two ostensibly unrelated studies bundled together to make best use of time. Participants were randomly assigned to play the prosocial (Lemmings) or violent (Lamers) game. After participants played the video game they were asked to fill out a questionnaire gauging their reactions to the game and, once they finished, the experimenter reached for the materials for the second study before knocking over a tin of pens placed equidistant from both the experimenter and participant. As with the previous experiments, the experimenter waited to see if the participant would help gather the spilt pens. Once the pens had been gathered the experimenter began the second study, after which participants were probed for suspicion and debriefed.

### Results and Discussion

Here we adopted the exact protocol reported by Greitemeyer and Osswald [11], that is, we used classic exemplars of violent and prosocial games, decreased the exposure time to 8 mins, and adopted the Session-Continues form of the pen-drop task, yet we were still unable to show any detrimental effect of violent games on prosocial behavior, *χ* ^2^ (1, *N* = 32) = 0.53, *p* = .716, *V* = .129.

Again, we conducted a series of t-tests to determine whether our video game stimuli differed on potential variables of interest. As with Experiment 2, we found no difference between the video game stimuli on frustration (*t*(30) = 0.88, *p* = .387), arousal (*t*(30) = 1.10, *p* = .282), or interest (*t*(30) = 0.80, *p* = .430).

The descriptive statistics in Table 3 show that the helping rates were very similar across game conditions. Even if the motives for violence in classic video games are less ambiguous than the motives for violence in contemporary video games, then it appears to have no bearing on prosocial behavior. We were unable to demonstrate that classic violent and prosocial games prime different rates of prosocial behavior, measured using the pen-drop task.

**Table 3:** Frequency of prosocial behavior across video game conditions in Experiment 3.

	Behavioral Outcome	
Video Game Condition	Help	No Help
Violent	11 (68.8)	5 (31.2)
Prosocial	9 (56.3)	7 (43.7)

Percentages in parentheses.

## General Discussion

Three experiments failed to find a detrimental effect of violent video games on prosocial behavior, despite using contemporary and classic games, delayed and immediate test-phases, and short and long exposures. While this study is not definitive evidence that violent video games have no detrimental effect on prosocial behavior, it might be that previously raised concerns regarding the impact of violent games on prosocial behavior may be mismatched or disproportionate. In this study, the context in which the prosocial task was administered had more influence over whether participants helped or not than did the type of video game they played. These findings may be viewed as being in line with previous research that has similarly failed to demonstrate a detrimental effect of violent video games on prosocial behavior (e.g. [26]).

Experiments 1 and 2 were conceptual replications, designed to extend the basic finding reported by Greitemeyer and Osswald [11] using contemporary video games, while Experiment 3 was a more precise replication using classic games. Across all three experiments we could not find a decrease in prosocial behavior. We followed suggestions by Greitemeyer and Osswald but it seems that previously intuitive, yet untested, ideas that longer exposures and contemporary games should elicit stronger effects on behavior do not hold.

We concede that our failure to find an effect may be due to the relatively small cell sizes reported in each experiment (16 participants per cell). We find this an unlikely reason for our failure to replicate, however, given that past research, including Greitemeyer and Osswald [11], used similar cell sizes [12], [13]. We further reject the poor power criticism of these experiments because in each the effect sizes are very small, ranging from 0 to.188. Indeed, in order to have sufficient cell sizes to reject any of the null hypotheses reported here, the number of participants needed in each experiment would range between 223 (for an effect size of.188, reported in Experiment 2) and 12 559 (for an effect size of.025, reported in Experiment 2). Not only would this be impractical but, given the small effect sizes, the applied value of any significant result would need to be called into question.

Other criticisms could be aimed at our choice of stimuli. First, we only selected single exemplars of contemporary video games to represent each game category. Other studies use multiple exemplars or even pilot test multiple exemplars, finally settling on two that score similarly on experience measures (e.g. interest, frustration, or arousal, see [22]). We defend our decision to use single exemplars by highlighting that the games we chose were both popular and commercially available at the time of data collection, which is where the value of this research lies. It remains possible, however, that unexamined unique characteristics of the games may have inhibited an effect of violent games on prosocial behavior [17], [18], [27]. Second, it is possible that our failure to find an effect of violent games is due to participants not recognizing the violent nature of the games. However, this seems highly unlikely. Grand Theft Auto IV is rated MA15+ according to the Australian Classification Board because it contains strong violence that is relatively frequent and strong in playing impact. Moreover, this game is used as a violent exemplar in other research [28], [29] with Chong and colleagues stating that the game world of Grand Theft Auto IV is “fraught with violence and players are rewarded and reinforced for their use of violence in order to advance in the game” (p.962) [29]. It remains possible, however, that the failure to demonstrate a benefit of playing prosocial games may be due to ambiguity of prosocial behavior in the selected prosocial game. It is not entirely clear whether World of Zoo is perceived as having prosocial content.

Further, we believe that the reported null findings are important, given that the current climate in social psychology is geared towards replication of classic findings (for a wide review of the current climate see [30] and associated commentaries, as well as [31]), with recent failures to replicate calling into question the legitimacy of widely regarded effects in social psychology [32]–[34]. It is well known that novel and surprising findings are more prone to publication bias [35] and likely to be false-positives [36]. Unfortunately, this leads to null results being viewed as less interesting because they are often unfairly labeled as “difficult to interpret” [31]. Of course, if null results are never reported, then we are only seeing a partial account of the true nature of any given effect. The Australian Government has even criticized research practices in the field for failing to include null effects in meta-analyses [37]. Given these pitfalls of scientific communication, methodical replication is paramount to the academic integrity of the field. We believe that our findings are a step in the right direction towards rebuilding that integrity.

Finally, there is some recent evidence showing that prosocial behavior towards strangers (compared to friends or family) is most strongly affected by violent video game habits (mediated by decreased empathic concern) [38]. Given that the experimenter had never met any participants prior to test, we can speculate that prosocial behavior should have been lower after playing a violent game. Considering this and our other attempts at creating optimal circumstances for the effect to reveal itself, we further speculate that the concern over the effect of violent video games is mismatched. Of course, Greitemeyer and Osswald [11] used multiple measures of prosocial behavior and the failure to replicate with one measure should not discredit their work. To this end, it is important that further work attempts to explore the effect that violent video games might have on prosocial behavior with multiple measures and different stimuli. However, it remains possible that, in terms of impact on prosocial behavior, public concern over violent video game play should be minimal.
